# Central Nervous System Mechanisms and Treatment Response in Chronic Ocular Surface Pain: Protocol for a Cross-Sectional Observational Phenotyping Study

**DOI:** 10.2196/84240

**Published:** 2026-01-15

**Authors:** Lindsey B De Lott, Steven E Harte, Chelsea Kaplan, David A Williams, Roni Shtein, Maria A Woodward, Alexander Tsodikov, Tatiana Deveney, Anat Galor, Anne Shea, Charles Schultz, Clare McKolay, Kathy A Scott, Daniel J Clauw

**Affiliations:** 1 Department of Ophthalmology and Visual Sciences University of Michigan Ann Arbor, MI United States; 2 Chronic Pain and Fatigue Research Center Department of Anesthesiology University of Michigan Ann Arbor, MI United States; 3 Biostatistics School of Public Health University of Michigan Ann Arbor, MI United States; 4 Bascom Palmer Eye Institute University of Miami Miami, FL United States; 5 Surgical services Miami Veterans Administration Medical Center Miami, FL United States; 6 Department of Neurology University of Michigan Ann Arbor, MI United States

**Keywords:** ocular pain, chronic pain, nociplastic, dry eye, protocol, observational study

## Abstract

**Background:**

Chronic ocular surface pain (COSP), occurring either in isolation or as part of numerous ocular conditions, such as dry eye syndrome, is a leading cause of eye care visits in the United States. Conventional treatments directed at the ocular surface—the perceived pain source—are often inadequate for pain relief. We hypothesize that some individuals with COSP are experiencing symptoms driven by central nervous system (CNS) dysfunction, similar to chronic overlapping pain conditions, rather than solely pathological problems in the eye. Some individuals with chronic overlapping pain conditions (eg, fibromyalgia) show evidence of nociplastic pain mechanisms, where the pain results from amplified or dysregulated CNS signaling and sensory processing. Although data exist suggesting the presence of nociplastic pain features in COSP, there is a need for comprehensive studies.

**Objective:**

Our aim is to rigorously define the role of nociplastic pain in COSP with a large, representative cohort of 200 participants with COSP using established clinical, neurobiological, and treatment response features. We propose that as sign and symptom discordance increases, features indicative of nociplastic pain will also increase.

**Methods:**

In aim 1, we will clinically phenotype participants with COSP, using validated patient-reported outcome measures and standard ocular exams. In aim 2, we will compare the neurobiological features of nociplastic pain across the discordance spectrum among a subset of aim 1 participants using multimodal evoked sensory testing (pressure, thermal, and visual testing) at sites both local to and remote from the eye. Participants will also complete structural and functional brain magnetic resonance imaging to assess regions important for pain perception and modulation. In aim 3, we will examine and validate predictors of COSP pain responses before and after application of a topical anesthetic to the ocular surface, which should block peripherally induced discomfort to allow for clarification of pain origination.

**Results:**

We received funding for this project in August 2024. Recruitment and enrollment began in January 2025 after protocol development and piloting were completed. This study is ongoing with 59 participants enrolled and 51 participants completing the study visits as of January 2026.

**Conclusions:**

Findings from the study have the potential to fundamentally change the way ocular pain syndromes are conceptualized, diagnosed, and treated. This work will not only help identify new CNS-directed pain treatments for a subset of patients with COSP but also help us better understand why many peripherally directed therapies are destined to be ineffective in a subset of individuals experiencing COSP.

**International Registered Report Identifier (IRRID):**

DERR1-10.2196/84240

## Introduction

Chronic ocular surface pain (COSP) encompasses a number of interrelated ocular pain symptoms, such as burning pain or eye irritation. Although COSP can occur as a symptom of several ocular conditions, including dry eye disease, it is well recognized that it can also occur in isolation. COSP is a leading cause of eye care visits in the United States, affecting more than 5% of people over their lifetime [1-3]. Because the symptoms are relentless and can be lifelong, patients experience decreased quality of life [4,5], similar to those with an immobilizing hip fracture or moderate to severe angina [6], decreased visual functioning [7,8], and higher odds of suicidal ideation [9].

A complex interaction of loss of tear film homeostasis and ocular surface inflammation (leading to pain [ie, nociceptive pain]) or pain arising from damaged corneal nerves (ie, neuropathic pain) drives pain symptoms in many individuals with COSP [10]*.* However, treatments aimed at restoring tear film homeostasis, reducing ocular inflammation, or even promoting tissue and nerve repair often do not relieve the pain for many people with COSP. Such is the case with the 10 currently recognized chronic overlapping pain conditions (COPCs)—a set of chronic pain conditions, including fibromyalgia, irritable bowel syndrome, temporomandibular disorders, chronic migraine, chronic tension type headache, chronic low back pain, chronic fatigue syndrome, vulvodynia, endometriosis, and interstitial cystitis or bladder pain syndrome [11]. In regional COPCs, such as temporomandibular disorder, interstitial cystitis, and bladder pain syndrome, a subset of individuals experiences peripherally mediated pain, as we know occurs in COSP. However, a prominent subset experiences centrally mediated nociplastic pain, where amplified or dysregulated neural signaling and sensory processing within the central nervous system (CNS) is now thought to be the primary or maintaining pathogenic pain mechanism [12-15]. In fact, these same nociplastic pain conditions are now referred to as “primary pain” conditions because the pain is the problem and not an underlying disease-causing damage or inflammation leading to pain. In nociplastic pain, therapeutic strategies that target the CNS are key to delivering pain relief.

We hypothesize that a subset of individuals with COSP experience symptoms driven primarily by CNS dysfunction rather than pathological problems exclusively with the eye. This premise is supported by observed nociplastic pain features among subsets of individuals with COSP [16], including (1) pain with minimal ocular surface signs (ie, sign and symptom discordance) [8,17], (2) multisite pain and CNS-mediated somatic symptoms (eg, fatigue) [18-20], (3) augmented CNS pain processing on evoked sensory testing and neuroimaging [21-23], and (4) persistent pain after topical anesthetic application [21]. However, no comprehensive studies have investigated clinical, neurobiological, and treatment response features of nociplastic pain in COSP*.* To address our hypothesis, we will conduct a cross-sectional, observational study to rigorously define the role of nociplastic pain in COSP (inclusive of dry eye disease but without structural, mechanical, and infectious causes), with a large, representative cohort of participants using established clinical phenotypic, neurobiological, and treatment response features.

## Methods

### Overview and Study Aims

This prospective, observational cohort study will recruit 200 individuals with COSP, with or without ocular surface signs (eg, dry eye syndrome but no other structural, mechanical, or infectious causes) from the University of Michigan (U-M). Our study design and selection of clinical and neurobiological measures are adapted from consensus methods developed as part of the National Institutes of Health (NIH) Helping to End Addiction Long-term (HEAL) initiative and several pain research networks [24-27].

The first aim of our project is to perform a clinical pain phenotyping study of participants with COSP along the sign and symptom discordance continuum. Participants will complete an ocular surface examination and standardized patient-reported outcome measures, including pain intensity assessment before and following application of topical anesthetic (proparacaine) to the ocular surface. On the basis of the ocular surface symptoms and signs, participants will be placed on a sign and symptom discordance continuum as described by Vehof et al [28]. This method has been used in observational studies and clinical trials [8,29].

The second aim of our project is to determine the neurobiological features of participants with COSP across the sign and symptom discordance continuum. A subset of 80 participants with COSP (discordant: n=40, 50%; concordant: n=40, 50%) and 40 controls (healthy controls: n=20, 50%; “positive” controls with a diagnosis of fibromyalgia: n=20, 50%) will also undergo multimodal quantitative sensory testing (QST), such as pressure, temperature, and visual modalities, and structural and functional magnetic resonance imaging (fMRI). We will also capture corneal subbasal nerve fiber density using in vivo corneal microscopy for exploratory analyses. Fibromyalgia was selected as a “positive” control as it is the prototypical nociplastic pain condition [12].

The final aim of our project is to integrate clinical and neurobiological data to determine which features predict response to a peripherally acting topical anesthetic. Our model of clinical predictors will be validated in 50 newly recruited participants with COSP. The clinical, neurobiological, and treatment response data will be integrated into our analyses to provide a robust, contextualized, and actionable interpretation of our results.

The SPIRIT (Standard Protocol Items: Recommendations for Interventional Trials) checklist is provided in Multimedia Appendix 1.

### Study Design and Site

Our study adopts an observational cross-sectional cohort design. This study will occur at a single center (U-M), leveraging the resources at the U-M Kellogg Eye Center (Department of Ophthalmology and Visual Sciences) and U-M Chronic Pain and Fatigue Research Center (CPFRC), Department of Anesthesiology.

### Ethical Considerations

This study has been approved by institutional review boards of the University of Michigan Medical School (HUM00256221). Participants will receive incentive payments upon completion of the study visits, including US $50 for the clinical assessment and US $150 for the neurobiological assessment. Informed consent is obtained either in person or over the phone after the details of the study and possible risks of the study are explained. All study procedures are in accordance with the ethical standards of the institutional review boards and with the Declaration of Helsinki. Data will be stored as discussed in the section on Data Management and Sharing.

### Study Participants

Individuals with COSP aged between 18 and 70 years will be invited to participate. For the first 2 aims of our study, recruitment of participants with COSP will end when 200 participants are enrolled. We will also recruit an additional 40 control participants, 20 (50%) healthy controls and 20 (50%) “positive” controls with a diagnosis of fibromyalgia. For the third aim of our study, we will recruit an additional 50 participants with COSP following initial analyses to validate our clinical phenotyping results.

Our study will use 2 sets of inclusion and exclusion criteria. The first set of criteria is for enrollment into the clinical phenotyping project for all participants (Textbox 1). The second set of criteria is necessary for the safe and valid conduct of the neurobiological testing protocol (subset of participants with COSP: n=80; control participants: n=40; Textbox 2).

**Inclusion and exclusion criteria for clinical phenotyping assessment.**

**Inclusion criteria**

- Provide a signed and dated informed consent form
- Willing to comply with all study procedures and be available for the duration of the study
- Adults aged between 18 and 70 years
- Ocular surface disease index score of 13 or greater
- Patients reporting ocular surface pain lasting 3 months or more, with pain present for more than half of those days
- Ability to read and speak English to allow for written informed consent and patient-reported outcomes measures

**Exclusion criteria**

- Adults older than 70 years (excluded due to potential vascular disease leading to abnormal functional imaging findings in older patients)
- Intraocular pressure lower than 5 mm Hg and higher than 22 mm Hg in each eye
- Current diagnosis of any eye infection or intraocular inflammation (uveitis)
- History of ocular herpetic keratitis
- Ocular surgery within 6 months (eg, cataract surgery)
- Previous corneal surgery (eg, laser-assisted in situ keratomileusis)
- Eyelid abnormalities that affect lid function (eg, ectropion, entropion, and lagophthalmos)
- Extensive ocular surface scarring or a condition that may compromise ocular surface integrity (eg, Stevens-Johnson syndrome)
- Current opioid use
- Diagnosed cognitive impairment, serious mental illness (eg, schizophrenia; anxiety and depression are not exclusions), or other condition that may impair decision-making capacity
- Pregnancy
- Persons in prisons
- Anything that would place the individual at increased risk or preclude the individual’s full compliance with or completion of the study
- Individuals receiving disability benefits or compensation within the past year or involved in litigation
- Any other diseases or conditions that would make a patient unsuitable for study participation, as determined by the principal investigator (including, but not limited to, severe psychiatric disorders, active suicidal ideations or history of suicide attempts, uncontrolled drug or alcohol addiction, autoimmune conditions on active biologics and immunosuppressive therapies, and visual or hearing difficulties precluding participation).

**Additional inclusion and exclusion criteria for the subset of participants undergoing neurobiological testing.**

**Additional inclusion criteria**

- Normal visual acuity or correctable (with corrective lenses, glasses, or contacts) to at least 20/40 for reading instructions in the magnetic resonance imaging (MRI) and visual sensitivity testing
- No contraindications to MRI (eg, metal implants)
- Willingness to refrain from pain medications, such as nonsteroidal anti-inflammatory drugs and acetaminophen, for 12 hours before neuroimaging and quantitative sensory testing
- Willingness to refrain from alcohol and nicotine on the day of quantitative sensory testing and neuroimaging (alcohol and nicotine consumption allowed after testing is completed)
- Willingness to refrain from physical activity or exercise that would cause muscle or joint soreness for 48 hours before testing (routine exercise or activity not leading to soreness is acceptable)
- Able to lie still on their back for 1 hour during MRI
- Completed all clinical testing, including a complete ocular exam and patient-reported outcome measures

**Additional exclusion criteria**

- Severe claustrophobia precluding MRI and evoked pain testing during scanning
- Unable to lie comfortably during MRI
- Current, recent (within the last 6 months), or habitual use of artificial nails or nail enhancements (artificial nails can influence pressure pain sensitivity at the thumbnail)
- Diagnosed or self-reported epilepsy because the visual testing strategy uses flashing lights
- Diagnosed or self-reported visual-evoked migraines because the visual testing strategy uses flashing lights
- Tattoos above the shoulders (interfere with MRI)

### Recruitment and Screening

The U-M Kellogg Eye Center clinics will be the primary site for participant identification and recruitment. Our U-M online active research studies website [30], established patient research registry (HUM00254693), and Michigan Medicine community advertisements will also be used for recruitment. These multiple strategies allow for recruitment beyond Kellogg Eye Center clinics and Michigan Medicine to reach a broader and more representative population of individuals with COSP. For identifying control participants, individuals participating in ongoing CPFRC studies who provided consent for future correspondence about additional research opportunities will be contacted by letter with study details and an opt-out form if they do not wish to be further contacted. Contact with all interested participants will be initiated by phone or in the clinic by a dedicated research coordinator. Potential participants will be screened using study forms. Study visits will be scheduled. Both the clinical and neurobiological assessments will take place in person and on separate days.

Our priority is to facilitate and encourage participation. When possible, we will schedule appointments on the same day as standard care appointments. Research appointments are scheduled through our electronic medical record system, and participants will receive both email and phone reminders. Questionnaires can be completed 24 hours before the appointment to decrease overall appointment times.

### Clinical Assessment

Participants will first complete a battery of questionnaires via the web-based Qualtrics electronic data capture system. These can be completed up to 24 hours before their scheduled visit. A structured medical and ophthalmic history will be obtained and an ophthalmologic examination will be conducted. Participants who provided consent but were later found to be ineligible will be considered screen failures and withdrawn.

Validated questionnaires will assess eye pain, pain in other parts of the body, nonpain CNS-mediated symptoms (eg, mood, cognitive functioning, and sleep), other measures of psychological functioning, and sociodemographic information (Table 1). Questionnaires were selected based on recommendations from the NIH HEAL Initiative Common Data Elements for adult chronic pain. A structured medical, ocular, and surgical history will be obtained using case report forms. Data on the duration of COSP, previous diagnoses potentially underlying COSP, systemic and ocular interventions (medications and procedures) used to treat COSP, and response to these interventions will be collected. Information about current medications used and family members with chronic pain conditions will also be collected.

**Table 1:** Key questionnaires for all participants.

Category and concepts		Measures
**Pain and sensory symptoms**		
	Multisite pain	Michigan Body Map (widespread pain index)
	Symptom severity	Symptom Severity Score from the American College of Rheumatology 2016 fibromyalgia criteria
	Neuropathic pain	painDETECT Neuropathic Pain Symptom Inventory-Eye
	Ocular surface symptoms	Ocular Surface Disease Index
	Other chronic pain conditions with a nociplastic basis	Chronic overlapping pain condition screener
	Chronic sensory symptoms	General Sensory Sensitivity Questionnaire-8
	Photophobia	Visual Light Sensitivity Questionnaire-8
	Pain duration and frequency	Two items adapted for the eye from the National Institutes of Health Research Task Force Minimum Dataset
	Pain self-efficacy	Pain Self-Efficacy Questionnaire
	Pain acceptance	Chronic Pain Acceptance Questionnaire
	Pain catastrophizing	Pain Catastrophizing Scale
	Pain interference, enjoyment, and general activity	Pain, Enjoyment of Life, and General Activity Scale-3
	Pain interference-specific activity	PROMIS SF^a^ version 1.0–Pain Interference 4a
	Pain intensity	PROMIS SF version 2.0 0–Pain Intensity 3a
	Pain severity	Pain Numeric Rating Scale
**Nonpain central nervous system–mediated symptoms**		
	Physical functioning or QoL^b^	PROMIS SF version 2.0–Physical Function 4a
	Sleep	PROMIS SF version 1.0–Sleep Disturbance 4a
	Depression	PROMIS SF version 1.0–Depression 4a
	Anxiety	PROMIS SF version 1.0–Anxiety 4a
	Fatigue	PROMIS SF version 1.0–Fatigue 4a
	Social roles and activities	PROMIS SF version 2.0–Ability to Participate 4a
	Memory and cognition	PROMIS SF version 2.0–Cognitive Function 4a
	Sleep duration	Single item of sleep duration adapted from the Pittsburgh Sleep Quality Index
	Dysautonomia	Composite Autonomic Symptom Score-31
**QoL**		
	Visual QoL	National Eye Institute Visual Functioning Questionnaire-9 and 2 pain subscale items
	Overall QoL	World Health Organization Quality of Life-brief version
**Social and psychological**		
	Substance use	Tobacco, Alcohol, Prescription Medication, and other Substance Use tool
	Optimism	Life Orientation Test-Revised
	Trauma	Brief Trauma Questionnaire
**Change measure**		
	Impression of change	Patient Global Impression of Change scale

^a^PROMIS SF: Patient-Reported Outcomes Measurement Information System Short Form.

^b^QoL: quality of life.

A structured ophthalmic examination will be conducted at the U-M Kellogg Eye Center. Briefly, we will collect the best corrected visual acuity, intraocular pressures, and pupillary assessment. The optic disc will also be assessed to ensure there is no evidence of optic disc edema to suggest elevated intracranial pressure, which can cause head pain, or an ongoing ophthalmic condition as a cause for COSP. Ocular surface measures were selected based on their use in clinical trials (eg, the Dry Eye Evaluation and Management study) [31] and routine eye examinations to maximize translation to clinical care. All participants will receive proparacaine hydrochloride 0.5%, a rapid-acting topical anesthetic instilled in the inferior fornix of each eye. Ocular pain intensity will be assessed before and 30 seconds after instillation using the Numeric Rating Scale (0-10; 0=no pain and 10=worst pain imaginable). Following the proparacaine challenge, participants will undergo Schirmer testing with an anesthetic.

### Neurobiological Assessment

A subset of participants (participants with COSP: n=80; control participants: n=40) will undergo a neurobiological assessment during a separate appointment at the CPFRC. Participants with COSP from across the sign and symptom discordance spectrum will be selected to participate. We will use a standard discordance scoring system generated from the clinical assessment data as described by Vehof et al [28] and used in previous studies [29]. During this visit, participants will undergo a battery of static and dynamic QST, in addition to a structural and fMRI of the brain. Briefly, our QST battery includes assessments of (1) corneal sensation detection and pain threshold testing using a commercially available gas esthesiometer (Brill Engines); (2) local and remote pressure pain thresholds measured at the forehead above the eye and the trapezius, respectively, by algometry (FPX 25; Wagner Instruments) and suprathreshold pressure pain sensitivity measured at the thumbnail bed (Multimodal Automated Sensory Testing System; Arbor Medical Innovations); (3) local and remote heat pain threshold testing at the forehead above the eye and forearm (QST.lab); (4) conditioned pain modulation using trapezius pressure pain threshold as the test stimulus and hand immersion in 10 °C water as the conditioning stimulus; (5) mechanical temporal summation of pain at the volar forearm and above the eye (PinPrick stimulators; MRC Systems GmbH); and (6) visual sensitivity to a flashing annular checkboard presented at multiple levels of brightness (Michigan Visual Aversion Stress Test; U-M).

The neuroimaging assessment includes 4 different magnetic resonance imaging (MRI) procedures, including resting state functional connectivity MRI, evoked pain fMRI using thumbnail pressure, evoked visual stimulation fMRI using a flashing annular checkboard, and voxel-based morphometry. Imaging data will be obtained on a 3.0 T General Electric MRI scanner located at the fMRI laboratory at U-M.

Finally, participants will undergo noninvasive, in vivo corneal confocal microscopy to obtain images of the subbasal corneal nerve fiber layer. The Heidelberg Retinal Tomograph II Rostock Cornea Module, a laser scanning confocal biomicroscope, provides 400 × 400 µm coronal images of the cornea. After placement of topical anesthetic eye drops and a moisturizing gel, contact examination will be performed to image the central cornea. Five representative still images of the central subbasal nerve plexus will be evaluated using image analysis software to quantify nerve density and morphology (eg, microneuromas). Images can be preprocessed using software incorporated in the instrument or NeuronJ, a plug-in of the NIH ImageJ analysis software. No gold standard exists for corneal image preprocessing. We will use whichever of these methods or any newly developed method with the best empirical support at the time the analyses are performed. Textbox 3 depicts key elements of the neurobiological testing protocol.

**Key elements of the deep phenotyping assessment for a subset of participants (participants with chronic ocular surface pain: n=80; controls: n=40).**

**Quantitative sensory testing**

- Pressure pain sensitivity
- Heat pain threshold
- Corneal sensation
- Visual sensitivity
- Conditioned pain modulation
- Temporal summation

**Neuroimaging**

- Structural magnetic resonance imaging (T1 and T2)
- Resting state functional magnetic resonance imaging (before and after evoked pain)
- Evoked pain functional magnetic resonance imaging

### Study Oversight

Study oversight will be under the direction of an independent study monitor (TD) and the principal investigator (LBDL). A study monitor was selected as an additional protection against risks for human participants. The study monitor will be available in real time to review and recommend appropriate actions regarding adverse events and other safety issues to the principal investigator and the study team.

### Statistical Design and Analysis

Our central hypothesis is that as the degree of symptoms out of proportion to signs (ie, discordance) increases, established clinical, neurobiological, and treatment response features indicative of nociplastic pain, such as multisite pain, multisensory hypersensitivity, and nonpain CNS-mediated symptoms, will also increase. We will use descriptive statistics to explore the data across aims, including cross-sectional correlation analyses of discordance with clinical and neurobiological data, as well as changes in pain intensity scores pre- and posttopical anesthetic application.

Likewise, linear regression models will be fit to assess the effects on the response variables, including discordance measures regressed on clinical nociplastic pain features, both before and after adding neurobiological features. Analysis of covariance–style linear regressions of change in pain intensity will be performed with nociplastic pain features, discordance, and pain intensity at baseline as covariates. For all models, the list of covariates will include systemic medications that potentially affect pain and ocular surface dryness treatment responses and other clinical and demographic variables. Backward variable selection procedures will be used along with Bayesian information criteria for best model selection. In high-dimensional settings, an elastic net regularization will be done to stabilize the regressions. Preference will be given to the least absolute shrinkage and selection operator penalties that assign 0 weights to nonsignificant predictors. Cross validation (10 fold) with 200 participants will be done to provide an approximate, unbiased assessment of model performance. This will be followed by second-order validation using an external validation set of 50 participants with COSP. Residuals will be examined for possible departures from normality. Generalized linear model and feature transformations will be used, if needed, to deal with violations of linear model assumptions. Simultaneous model-based CIs will be reported that characterize the joint character of hypothesis testing rather than a multiple comparisons adjustment.

Logistic models will be used with dichotomous outcomes for discordance (yes or no) in aims 1 and 2 and 50% relative improvement in pain intensity (yes or no) in aim 3 (including a mixed logistic generalized linear mixed model in the longitudinal analytic setup). Linear mixed models (or generalized linear mixed models) will be used as an alternative in aim 3. Gaussian random intercept shared within the participant will be used to model the participant-specific effect, with measurements before and after treatment serving as the response. The model will be used to characterize the variance components. Estimates will be obtained by maximum likelihood, followed by best model selection and hypothesis testing using Bayesian information criteria and likelihood ratio model–based tests.

We will perform 2 exploratory analyses. First, a latent class or latent variable model will be used to explore an unsupervised measurement of the CNS involvement status (ie, nociplastic pain). This will allow us to suggest an alternative to defining discordance as an empirical measure. Second, in a separate exploratory exercise, we will reconcile the results of this project with historical CPFRC studies of other chronic pain cohorts (eg, chronic low back pain and knee osteoarthritis) that explored CNS pain amplification and persistent pain after surgery. Intuitively, we anticipate general common features of CNS involvement across chronic pain conditions and will look for a possible joint model of multiple disease cohorts to assess disease-specific versus general features that characterize nociplastic pain.

Reasons for missingness will be analyzed using multinomial logistic regression. The results of formal missing data imputation by predictive-matching algorithms will be compared to the results of missing data exclusion in a sensitivity analysis. We do not expect the fraction of missing data to exceed 10%; therefore, 1 round of imputation will likely be sufficient. An alternative approach is multiple imputation [32].

### Power Calculations

We will be able to reliably detect a correlation (*r*) of 0.2 or higher with 81% power when tested at the 5% significance level with 200 participants. Higher correlations (*r*=0.40) were observed in our previous knee osteoarthritis studies between pain and the widespread pain index (included in the Michigan Body Map), measuring CNS amplification [33]. Published analyses of discordance in patients with dry eye disease based on an objective tear production measure (Schirmer tests) confirm effects at least as strong as in this study [8]. In the QST studies, the observed correlation was 0.2 between the forehead and forearm hot and cold pain intensity measurements and discordance measures [17]. Correlations of 0.25 were observed in the literature between burning ocular pain and the presence of hypersensitivity to heat in the forearm [34]. For the treatment response study (aim 3), 92% power can be obtained with as few as 40 participants with nociplastic pain, which is easily attainable with the sample size of 200 in our study. This assessment is based on the 2-tailed *t* test comparing pain intensity among participants with and without persistent pain in a study of 224 veterans (mean 6.7, SD 2.6 vs mean 4.5, SD 2.9) [21].

### Data Management and Sharing

Data will be stored on Health Insurance Portability and Accountability Act (HIPAA)–compliant systems (eg, [REDCap; Research Electronic Data Capture; Vanderbilt University]) to protect the personal health information of participants. Participant demographics will be collected using NIH-endorsed common data elements, for example, age, sex, race, and ethnicity. Participant clinical data will be collected using forms from the NIH Common Data Elements repository when possible [35]. Some participant-reported outcome data (eg, Patient Reported Outcomes Measurement Information System 29; version 2) have standard collection forms available in the NIH Common Data Elements repository. Others that do not (eg, Ocular Surface Disease Index) will be collected using standard survey methodology procedures.

Sufficient data from this project will be preserved to enable validation and replication of the research findings described in the aims. Deidentified data, including participant clinical data, sensory testing data, and survey responses, will be shared. To protect participant privacy and confidentiality, shared data will be deidentified using the “safe harbor” method, in compliance with the HIPAA privacy rule. We will archive our corneal image data in the Deep Blue Data (DBD) repository. DBD is a generalist repository developed at the U-M in support of sharing and preserving research data. DBD provides free public access and downloading of data. All other data, including neuroimaging data, will be deposited in the National Institute of Mental Health Data Archive (NDA). NDA makes human participants’ data collected from hundreds of research projects across many scientific domains available. NDA provides infrastructure for sharing research data, tools, methods, and analyses, enabling collaborative science and discovery. Data deposited in DBD will be accessible for at least 10 years, after which they will be appraised for continued preservation by the university library personnel. Data deposited in the NDA will be archived indefinitely.

## Results

Our study is supported by a grant from the National Eye Institute (NIH R01EY036357). Recruitment and enrollment commenced in January 2025 following finalization and piloting of the study protocol. As of January 2026, we have enrolled 59 participants and completed 51 clinical phenotyping study visits. Of those 51 participants, 15 have completed neurobiological phenotyping visits. The remaining participants are enrolled but have not completed either study visit.

## Discussion

### Expected Outcomes and Future Directions

The transfer of experience and knowledge from other COPCs with a nociplastic component (eg, fibromyalgia, interstitial cystitis or bladder pain syndrome, and irritable bowel syndrome) to studies of the eye has strong potential to change how we treat COSP and will advance our understanding of ocular pain mechanisms more broadly. This is the first-of-its-kind comprehensive study of clinical and neurobiological nociplastic pain features in COSP using validated, leading-edge pain assessments. The proposed work will open a new view of COSP as part of a broader systemic chronic pain state driven preferentially by CNS dysfunction in many patients. Our extensive clinical and neurobiological data can be leveraged to (1) support larger studies that validate our findings in broader populations, (2) design clinical criteria for detecting subsets of patients with COSP with nociplastic pain, (3) identify those who may respond preferentially to CNS-directed therapies and monitoring both clinical and neurobiological responses to treatment, and (4) identify and modify antecedents of nociplastic pain development in patients with COSP. Ultimately, successful future treatment strategies for COSP depend not only on the success of addressing relevant nociceptive and neuropathic pain but also on the degree to which nociplastic pain can be identified and managed.

### Conclusions

COSP is disabling for many patients, and our current treatments aimed at the ocular surface—the presumed origin of pain—are insufficient. Advancing our understanding of pain mechanisms in COSP is critically needed to target treatments effectively. This research will rigorously define the role of nociplastic pain in COSP with a large representative cohort of patients using established clinical phenotypic, neurobiological, and treatment response features. This represents a crucial step forward in reaching the ultimate goal of realizing targeted therapeutic strategies tailored to individual patients experiencing COSP.

## Appendix

Multimedia Appendix 1 SPIRIT checklist.

## Abbreviations

CNS: central nervous system
COPC: chronic overlapping pain condition
COSP: chronic ocular surface pain
CPFRC: Chronic Pain and Fatigue Research Center
DBD: Deep Blue Data
fMRI: functional magnetic resonance imaging
HEAL: Helping to End Addiction Long-term
HIPAA: Health Insurance Portability and Accountability Act
MRI: magnetic resonance imaging
NDA: National Institute of Mental Health Data Archive
NIH: National Institutes of Health
QST: quantitative sensory testing
SPIRIT: Standard Protocol Items: Recommendations for Interventional Trials
U-M: University of Michigan

## Data Availability

The datasets generated during this study will be available in the Deep Blue Data and National Institute of Mental Health Data Archive repositories.
